# Outcomes of primary versus revisional robotically assisted laparoscopic Roux-en-Y gastric bypass: a multicenter analysis of ten-year experience

**DOI:** 10.1007/s00464-020-08061-x

**Published:** 2020-10-07

**Authors:** Pouya Iranmanesh, John Fam, Thomas Nguyen, David Talarico, Kavita D. Chandwani, Kulvinder S. Bajwa, Melissa M. Felinski, Leon V. Katz, Sheilendra S. Mehta, Stephan R. Myers, Brad E. Snyder, Peter A. Walker, Todd D. Wilson, Angielyn R. Rivera, Connie L. Klein, Shinil K. Shah, Erik B. Wilson

**Affiliations:** 1 Division of Minimally Invasive and Elective General Surgery, Department of Surgery, McGovern Medical School, University of Texas Health Science Center at Houston, 6431 Fannin Street, Houston, TX 77030 USA; 2 Weight Loss Surgery and Wellness Center, Tower Health, Reading Hospital, Reading, PA USA; 3 Texas Laparoscopic Consultants, Houston, TX USA; 4 Health First Medical Group, Rockledge, FL USA; 5 Michael E. DeBakey Institute for Comparative Cardiovascular Science and Biomedical Devices, Texas A&M University, College Station, TX USA

**Keywords:** Robotic surgery, Gastric bypass, Revisional bariatric surgery

## Abstract

**Background:**

Postoperative morbidity after laparoscopic bariatric surgery is considered higher for patients undergoing revisional versus primary procedures. The objective of this retrospective cohort study was to compare outcomes between patients undergoing primary versus revisional robotically assisted laparoscopic (RAL) Roux-en-Y gastric bypass (RYGB).

**Methods:**

Data of all patients who underwent RAL primary and revisional RYGB between 2009 and 2019 at two accredited, high-volume bariatric surgery centers—the Memorial Hermann – Texas Medical Center, Houston, TX, and the Tower Health, Reading Hospital, Reading, PA, were analyzed. Primary outcomes were early (< 30 days) and overall postoperative complications. Secondary outcomes included intraoperative complications, operative times, conversions to laparotomy, length of hospital stay, early (< 30 days) postoperative readmissions and deaths.

**Results:**

Data of 1072 patients were analyzed, including 806 primary and 266 revisional RAL RYGB procedures. Longer operative times (203 versus 154 min, *P* < 0.001), increased number of readmissions for oral intolerance (10.5% versus 6.7%, P = 0.046) and higher rate of gastrojejunal stricture (6.4% versus 2.7%, P = 0.013) were found in the revisional group. Gastrointestinal leak rates were 0.2% for the primary versus 1.1% for the revisional group (*P* = 0.101). Early (< 30 days) reoperations rates were 2.2% for the primary versus 1.1% for the revisional group (*P* = 0.318). There were no statistically significant differences between groups in overall and severe complication rates.

**Conclusion:**

Patients undergoing RAL primary and revisional RYGB had comparable overall outcomes, with a non-significant higher early complication rate in the revisional group. Despite the study being underpowered to detect differences in specific complication rates, the morbidity seen in the revisional RYGB group remains markedly below literature reports of revisional laparoscopic RYGB and might suggest a benefit of robotic assistance. Further prospective studies are needed to confirm these results.

Bariatric surgery is currently established as the most effective treatment for morbid obesity and its related comorbidities [1, 2]. The advent of laparoscopy in the early 1990s has significantly improved perioperative outcomes for patients with obesity and has naturally emerged as the standard approach for most bariatric procedures [3–5]. The overall number of bariatric surgeries has constantly been increasing in the past decade across the world, with almost 686,000 procedures performed in 2016, including 51,000 (7.4%) revisional procedures [6]. The same data show that laparoscopic Roux-en-Y gastric bypass (RYGB) is the second most commonly performed bariatric surgery worldwide after the laparoscopic sleeve gastrectomy (SG), with 191,000 and 341,000 procedures, respectively.

In the US, 42,800 RYGB were performed in 2018, representing 17% of all bariatric surgeries [7]. Severe early complications after primary laparoscopic RYGB are relatively uncommon nowadays, with anastomotic leak rates ranging between 0.1 and 1.2% [8–11]. Higher morbidity and mortality have however been described after laparoscopic revisional RYGB, with anastomotic leak rates ranging from 4.5 to 11.8% [11–19].

Robotically assisted laparoscopic (RAL) surgery offers better visualization, articulated instruments, tremor filtration, ergonomic position and the possibility of performing handsewn anastomoses similarly to open surgery [20, 21]. Although little randomized data are available, outcomes after primary RAL bariatric surgery seem to be at least equivalent to the traditional laparoscopic approach. Some larger series have found lower anastomotic leak and stricture rates when using the robotic platform for primary RAL RYGB [22–24]. Smaller studies have suggested the same benefits for patients undergoing revisional RAL RYGB [25–27]. The objective of this study was to compare outcomes between patients undergoing primary versus revisional RAL RYGB. The hypothesis was that both groups would have similar early and late postoperative complication rates.

## Material and methods

### Design, setting and participants

This study was a retrospective analysis of a prospective database containing all patients > 18 years who underwent RAL primary or revisional RYGB between 2009 and 2019 at two accredited bariatric surgery centers: The University of Texas Health Science Center at Houston and Memorial Hermann – Texas Medical Center (Houston, TX), and Tower Health, Reading Hospital (Reading, PA).

The primary RYGB group included patients who had never undergone any previous bariatric or antireflux surgery. The revisional RYGB group included patients undergoing any of the following surgeries:

- Conversion from any other previous bariatric procedure to RYGB
- Revision of an existing RYGB
- RYGB performed for weight loss purposes in patients who had undergone previous antireflux procedures

Patients undergoing revision of an existing RYGB were included only if at least one anastomosis was revised. Patients undergoing RYGB for purposes other than weight loss (i.e., gastroesophageal reflux control) were excluded.

Surgeons were classified according to their experience in robotic bariatric surgery as senior (> 200 RAL RYGB) and junior (< 200 RAL RYGB). All surgeons had performed at least 100 RAL general surgery procedures and 50 primary RAL RYGB and had a minimum of 2 years of bariatric experience beyond their fellowship training.

### Technique

All surgeries were performed using the Si or Xi version of the da Vinci® Surgical System (Intuitive Incorporation, Sunnyvale, CA, USA). The same surgical technique was used in both institutions. All gastrojejunal anastomoses were performed side-to-side using a fully handsewn technique with absorbable suturing material. A bougie was used to calibrate the gastrojejunal anastomosis to approximately 1.5 cm final stomal diameter. All jejunojejunal anastomoses were performed in a side-to-side fashion using a 60-mm endoscopic linear stapler and enterotomy defects were closed using a handsewn technique with absorbable suturing material or endoscopic linear stapler as well. Details of our operative technique used for RAL RYGB have been previously published elsewhere [28].

### Collected data

Baseline characteristics included age, gender, body mass index (BMI) at the time of surgery, comorbidities, history of previous abdominal surgeries and length of follow-up after surgery. For the revisional group, types of conversion or revision were also collected. Primary outcomes were early (< 30 days after surgery) and overall postoperative complications. Overall postoperative complications included all complications occurring at any time between surgery and end of patient follow-up. Secondary outcomes were intraoperative complications, operative times, conversions to laparotomy, length of hospital stay (LOS), early (< 30 days after surgery) readmissions and deaths. Postoperative complications were ranked according to the Dindo–Clavien classification [29], with severe complications defined by a score of ≥ IIIa (complications requiring percutaneous, endoscopic or surgical intervention, and/or intensive care unit management). To ensure data completeness and accuracy, electronic records of each patient were reviewed by the primary author between February and August 2019 prior to performing statistical analyses. All readmissions, reoperations, postoperative endoscopies as well as endoscopic and radiological interventions were tracked manually and added to the database where appropriate.

### Statistical analysis

All calculations were performed using PASW Statistics 18 (IBM Corporation, Armonk, NY). Outcomes between groups were compared using Mann–Whitney and Fisher’s exact tests where appropriate. Subgroup analyses according to length of follow-up (> versus < 12 months) and according to surgeon’s experience (senior versus junior) were additionally performed using the same statistical tests. To assess potential confounding factors, baseline characteristics predicting early and overall complications with a *P*-value < 0.1 on univariate analysis were subsequently entered into a multivariate analysis based on a logistical regression. A two-sided *P*-value ≤ 0.05 was considered statistically significant.

### Ethical and quality considerations

All patients gave written consent to have their data collected and used anonymously for research purposes. This study was approved by the Institutional Review Boards of the University of Texas Health Science Center at Houston, Texas and Tower Health, Reading Hospital, Pennsylvania. Reporting of results was based on the Strengthening the Reporting of Observational Studies in Epidemiology (STROBE) Statement.

## Results

Data of 1079 patients were analyzed. Seven patients were excluded, including 5 patients who underwent revisional RYGB without anastomotic revision and 2 patients who underwent unplanned sleeve gastrectomy instead of RYGB due to intraabdominal adhesions. A total of 1072 patients were included in the study. Among them, 806 underwent primary and 266 revisional RAL RYGB, including 467 primary and 237 revisional procedures at Memorial Hermann – Texas Medical Center, and 339 primary and 29 revisional procedures at Tower Health – Reading hospital, respectively. Baseline characteristics are shown in Table 1. Patients undergoing revisional RYGB were significantly older and had a lower BMI at time of surgery. Obesity-related comorbidities were more prevalent in patients undergoing primary RYGB, except for GERD which was more prevalent in the revisional group. All patients had a minimum postoperative follow-up of 3 months, with a significantly longer mean follow-up for the revisional group (17.3 versus 16.6 months, *P* < 0.001). Types of revisional procedures are detailed in Table 2. Among the 125 patients with conversion from adjustable gastric bands, 86 underwent a single-stage and 39 underwent a two-stage procedure.

**Table 1:** Baseline characteristics

	Primary (*n* = 806)	Revisional (*n* = 266)	*P*-value
Age, mean (SD), years	45.4 (11.9)	50.3 (10.9)	** < 0.001**
Women-to-men ratio (F:M)	629:177 (3.6:1)	227:39 (5.8:1)	**0.011**
BMI, mean (SD), kg/m^2^	45.9 (16.5)	41.3 (9.1)	** < 0.001**
Comorbidities			
- Hypertension (%)	494 (61.3)	122 (45.9)	** < 0.001**
- T2D (%)	362 (44.9)	76 (28.6)	** < 0.001**
- Dyslipidemia (%)	354 (43.9)	76 (28.6)	** < 0.001**
- Gastroesophageal Reflux Disease^a^ (%)	442 (54.8)	166 (62.4)	**0.032**
- Obstructive sleep apnea^b^ (%)	363 (45)	71 (26.7)	** < 0.001**
Previous abdominal surgery (%)	521 (64.6)	156 (58.6)^c^	0.092
Length of follow-up, mean (SD), months	16.6 (± 20)	17.3 (± 19.7)	** < 0.001**
Patients with follow-up ≥ 12 months (%)	427 (53.0)	133 (50.0)	0.436

*P*-values in bold indicate statistically significant results

*SD *standard deviation, *BMI* body mass index, *T2D* Type 2 Diabetes

^a^Based on typical symptoms, use of proton pump inhibitor medication and/or endoscopic findings

^b^Based on typical history and/or polysomnography results

^c^156 patients who previously underwent another abdominal surgery besides the index procedure defining revisional status

**Table 2:** Types of revisions

Initial surgery	*n*	%
*Other bariatric procedures (conversions)*	230	86.4
- AGB	125	47.0
- VBG	44	16.5
- SG	39	14.7
- Fixed Molina band	15	5.6
- Transoral gastroplasty	6	2.2
- One-anastomosis gastric bypass	1	0.4
*RYGB (revisions*)	24	9.0
*Antireflux procedures*	10	3.8
- Nissen fundoplication	6	2.2
- Hill repair	2	0.8
- Toupet fundoplication	1	0.4
- Transoral incisionless fundoplication	1	0.4
*Other*	2	0.8
- Lateral gastric plication	1	0.4
- Unclear type of fundoplication	1	0.4

The denominator for all lines is the total cases of revisional procedures (*n* = 266)

*AGB* adjustable gastric band, *VBG* vertical banded gastroplasty, *SG* sleeve gastrectomy, *RYGB* Roux-en-Y gastric bypass

Comparison of primary and secondary outcomes is shown in Table 3. There were no differences between groups in terms of early (< 30 days) and overall postoperative complication rates. Early and overall severe (grade ≥ IIIa) complication rates were also similar between groups. There were no differences between groups in early and overall complication rates on univariate and multivariate regression analyses according to baseline characteristics. Subgroup analyses according to length of follow-up and surgeons experience did not show different results. Detailed complication breakdown can be seen in Table 4. When analyzing each type of complication separately, the revisional group had significantly higher rates of gastrojejunal stricture and oral intolerance requiring readmission. All patients readmitted with oral intolerance were treated conservatively with intravenous fluids and antiemetics (grade I). There was no difference between groups for other types of complication.

**Table 3:** Primary and secondary outcome comparison

Outcomes	Primary (*n* = 806)	Revisional (*n* = 266)	*P*-value
Early (< 30 days) complications (%)	92 (11.4)	42 (15.8)	0.069
Early (< 30 days) severe complications^a^ (%)	28 (3.5)	12 (4.5)	0.456
Early (< 30 days) reoperations (%)	18 (2.2)	3 (1.1)	0.318
Overall complications^b^ (%)	227 (28.2)	87 (32.7)	0.163
Overall severe complications^a,b^ (%)	112 (13.9)	44 (16.5)	0.316
Overall reoperations (%)	79 (9.8)	25 (9.4)	0.905
Readmissions within 30 days (%)	67 (8.3)	30 (11.3)	0.174
Intraoperative complications (%)	6 (0.6)	4 (1.5)	0.237
Operative times, mean (SD), min	154 (± 51)	203 (± 78)	** < 0.001**
Conversions to laparotomy (%)	3 (0.4)	1 (0.4)	0.999
Length of hospital stay, mean (SD), days	3.4 (± 5.1)	3.1 (± 2.7)	0.866
Deaths (%)	2 (0.2)	0	0.999

*P*-values in bold indicate statistically significant results

*SD* standard deviation

^a^Defined by a Dindo–Clavien score ≥ IIIa

^b^Include all complications occurring during the total length of follow-up

**Table 4:** Complication breakdown

Type of complication	Primary (*n* = 806)		Revisional (*n* = 266)		*P*-value
	*n*	%	*n*	%	
Gastrointestinal leaks	2	0.2	3	1.1	0.101
- Gastrojejunal anastomosis	1	0.1	1	0.4	0.435
- Jejunojejunal anastomosis	0	0	1	0.4	0.248
- Gastric pouch staple line	0	0	1	0.4	0.248
- Gastric remnant staple line	1	0.1	0	0	0.999
Marginal ulcer	55	6.8	21	7.9	0.582
Requiring anastomotic revision	3	0.4	1	0.4	0.999
Gastrojejunal stricture	22	2.7	17	6.4%	**0.013**
Requiring anastomotic revision	0	0	2	0.8	0.061
Perforated gastrojejunal ulcer	14	1.7	1	0.4	0.134
Perforated jejunojejunal ulcer	1	0.1	0	0	0.999
Oral intolerance requiring readmission	54	6.7	28	10.5	**0.046**
Internal hernia	32	4.0	11	4.1	0.859
Jejunojejunal obstruction	5	0.6	0	0	0.341
Gastrogastric fistula	0	0	1	0.4	0.248
Small bowel obstruction	10	1.2	4	1.5	0.757
Incisional hernia	11	1.4	0	0	0.075
Esophageal ulcer bleed	0	0	1	0.4	0.248
Unlocalized gastrointestinal bleed	9	1.1	0	0	0.123
Iatrogenic colon injury	1	0.1	0	0	0.999
Ileum necrosis	0	0	1	0.4	0.248
Esophagogastric junction stricture	0	0	1	0.4	0.248
Intraabdominal fluid collection	1	0.1	1	0.4	0.435
Superficial wound infection	2	0.2	1	0.4	0.575
Unexplained, chronic abdominal pain	40	5.0	11	4.1	0.740
Negative diagnostic laparoscopy	15	1.9	5	1.9	0.999
Thromboembolic events	4	0.5	2	0.8	0.642
Pulmonary embolus	2	0.2	0	0	0.999
Deep veinous thrombosis	2	0.2	2	0.8	0.258
Pneumonia	5	0.6	1	0.4	0.999
Atelectasis	3	0.4	0	0	0.999
Myocardial infarction	1	0.1	1	0.4	0.435
Heart failure	1	0.1	0	0	0.999
Nephrolithiasis	3	0.4	0	0	0.999
Urinary tract infection	10	1.2	1	0.4	0.310
Acute renal failure	0	0	1	0.4	0.248
Congestion and fever	1	0.1	0	0	0.999

*P*-values in bold indicate statistically significant results

Severe early (< 30 days) postoperative complications (grade ≥ IIIa) and their management are shown in Table 5. The revisional group had a longer mean operative time (203 versus 154 min, *P* < 0.001). Rates of readmissions, intraoperative complications, conversions to laparotomy and mean LOS were similar between groups.

**Table 5:** Early severe postoperative complication breakdown and management

Type of complication	*n*	Grade	Management
*Primary group*			
Gastrojejunal anastomosis leak	1	IV	Laparoscopic washout and drainage, ICU management
Gastric remnant staple line leak	1	IIIb	Laparoscopic washout and drainage, gastrostomy tube insertion
Bleeding marginal ulcer	1	IIIa	Endoscopic hemostasis, PPI and sucralfate
Gastrojejunal anastomosis stricture	4	IIIa	Endoscopic balloon dilatation
Jejunojejunal anastomosis obstruction	5	IIIb	Laparoscopic jejunojejunal anastomosis revision
Incarcerated incisional hernia	4	IIIb	Laparoscopic or open incisional hernia repair
Internal hernia	2	IIIb	Laparoscopic reduction and closure of defects
Small bowel obstruction (early adhesions)	4	IIIb	Laparoscopic lysis of adhesions
Iatrogenic colon perforation	1	IV	Open colon resection, ICU management
Intraabdominal fluid collection	1	IIIa	Interventional radiology drain insertion
Large ureteral lithiasis	1	IIIb	Lithotripsy under general anesthesia
Heart failure	1	IV	ICU management
Myocardial infarction	1	IV	ICU management and PTCA
Bilateral pulmonary emboli	1	IIIa	Percutaneous catheter thrombolysis and anticoagulation
*Revisional group*			
Gastrojejunal anastomosis leak	1	IIIb	Laparoscopic washout and drainage
Jejunojejunal anastomosis leak	1	IIIa	Interventional radiology drain insertion
Gastric pouch staple line leak	1	IIIa	Interventional radiology drain insertion
Gastrojejunal anastomosis stricture	4	IIIa	Endoscopic balloon dilatation
Internal hernia	1	IIIb	Laparoscopic small bowel resection and closure of defects
Small bowel obstruction (early adhesions)	1	IIIb	Laparoscopic lysis of adhesions
Bleeding esophageal ulcer	1	IIIa	Endoscopic hemostasis and PPI
Myocardial infarction	1	IV	ICU management and PTCA
Aspiration pneumonia	1	IV	ICU management with mechanical ventilation and antibiotics

Early is defined by < 30 days after surgery. Complications are graded according to the Dindo–Clavien classification. Severe complications are defined by a grade ≥ IIIa

*ICU* intensive care unit, *PPI* proton pumb inhibitors, *PTCA* percutaneous transluminal coronary angioplasty

Intraoperative complications in the primary group included 2 positive gastrojejunal anastomosis air leak tests (managed with interrupted sutures), 1 misfired stapler load on the gastric remnant (managed by a running suture), 1 liver bleed (managed with electrocautery and hemostatic agents), 1 mesenteric bleed (managed by conversion to laparotomy and vessel ligation) and 1 patient who developed hyperkalemia with T-waves during general anesthesia (managed by insulin and calcium gluconate injections). Intraoperative complications in the revisional group included 3 small bowel injuries (managed by resection and anastomosis) and 1 pneumothorax (managed by pleural drain insertion). Conversion to laparotomy was necessary in three patients in the primary group (due to uncontrolled mesenteric bleeding, intestinal malrotation and small bowel injury, respectively) and one patient in the revisional group (due to small bowel injury). There were no conversions to standard laparoscopy. In the primary group, there was one early death on the first postoperative day due cardiac arrhythmia with asystole and a late death three years after surgery due to an internal hernia with extensive small bowel necrosis and septic shock, who died despite emergency surgery. There were no deaths in the revisional group.

## Discussion

To the best of our knowledge, this article describes the largest cohort of patients undergoing revisional RAL RYGB in the literature (*n* = 266). In this study, patients undergoing RAL primary and revisional RYGB had similar outcomes in terms of overall postoperative complication rates. The incidences of specific complications such as leaks, marginal ulcer or internal hernia were similar between groups, except for a higher rate of gastrojejunal strictures and readmissions for oral intolerance among the revisional group.

Differences between groups in baseline characteristics were most likely due to patients undergoing revisional surgery at least a few years after the initial procedure. At that time, patients usually had a lower BMI and varying degree of comorbidities remission, especially if they underwent surgery for complications such as persistent nausea or dysphagia. The higher prevalence of preoperative GERD in the revisional group could be explained by the natural postoperative history of procedures such as vertical banded gastroplasty, gastric band placement and sleeve gastrectomy, which have all been shown to cause de novo or worsening of GERD postoperatively in some patients [30–32] and for whom RYGB provides excellent results in terms of postoperative reflux control [32, 33]. Although statistically significant, the small mean difference in lengths of follow-up between groups (0.7 months) is unlikely to have clinical significance. Univariate and multivariate regression analyses were performed to adjust for these differences, without modifying the initial results.

Gastrointestinal leaks are a dreaded complication in bariatric surgery. Previous studies have suggested a lower leak rate after primary RAL RYGB compared to laparoscopic RYGB [22–24]. When considering all types of leaks, the incidence was only 0.2% (2/806) for the primary group and 1.1% (3/266) for the revisional group (*P* = 0.101). Among them, gastrojejunal anastomotic leaks rates were 0.1% (1/806) and 0.4% (1/266), respectively (*P* = 0.435). Leak rates in the literature range from 0.1% to 1.2% after primary laparoscopic RYGB [8–11] and 4.5 to 11.8% after revisional laparoscopic RYGB [11–19]. While the gastrointestinal leak rate found after primary RAL RYGB in this study (2/806, 0.2%) was consistent with the lowest leak rates found in the literature, the incidence of leaks after revisional RYGB (3/266, 1.1%) was markedly below what has been reported with the laparoscopic approach, suggesting an advantage of robotic assistance for revisional RYGB. Potential explanations for this difference are better visualization, increased mobility provided by articulated instruments and a more ergonomic position at the robotic console, possibly leading to a more precise dissection, better tissue handling and less surgeon fatigue during these procedures where extensive lysis of adhesions is often required. This more precise dissection could lead to improved maintenance of blood supply to tissues in an often hostile reoperative field. The possibility to perform fully handsewn anastomoses comparable to open surgery could be another factor, which has been analyzed for primary RYGB in other studies [22, 24]. Surgeon’s experience with robotically assisted bariatric surgery certainly played a role as well. Buchs et al. found that only 14 cases were necessary to overcome the learning curve for RAL RYGB [34]. In their systematic review, Pernar et al. found that less than 100 cases were sufficient to achieve plateau performance for most general surgery subspecialties [35]. All surgeons involved in this study had an experience of at least 150 RAL procedures, but there was no difference in outcomes between senior and junior surgeons.

When analyzing each type of complication separately, the only statistically significant differences were higher rates of readmissions for oral intolerance and gastrojejunal strictures in the revisional group. Oral intolerance was a minor complication in all patients, requiring only IV fluids and antiemetics (grade I). Higher rates of marginal ulcer and gastrojejunal stricture have been described previously after revisional RYGB [16]. Possible mechanisms include decreased micro-vascularization in tissue that has had previous surgery, which may lead to an exaggerated scar response. Macro-vascularization, i.e., left gastric artery perfusion, appears to be preserved, as evidenced by near equivalent leak rates in the revisional group. In this study, all gastrojejunal strictures were successfully treated by endoscopic balloon dilatation in both groups (grade IIIa), except for 2/17 patients from the revisional group who required surgical management (grade IIIb). This trend towards higher rate of surgical revisions for gastrojejunal stricture in patients undergoing revisional RYGB was however not significant. Of note, there was no difference between groups in rates of marginal ulcers and number of patients requiring gastrojejunal anastomosis revision due to refractory marginal ulcer.

The relatively high number of overall postoperative complications is most likely due to the thorough tracking of all postoperative adverse events, even minor complications. Moreover, complications were collected as far as 10 years after surgery for patients with longer follow-ups. The rates of severe complications, including early (< 30 days) severe complications and reoperations, were however comparable to previously published studies [16, 36, 37].

In this study, conversion to laparotomy was necessary in only 0.4% of patients in both groups (3/806 and 1/266, respectively). This rate is especially low for patients undergoing revisional RYGB compared to previous reports of conversion rates as high as 14% [27, 28].

This study has several limitations. It is a retrospective data analysis and not a randomized or prospective study. To reduce reporting biases, all patient records were however manually verified, including endoscopic, radiological and surgical reports. Even though no statistical difference was found between groups in terms of gastrointestinal leak rates (0.2% vs 1.1%, *P* = 0.101), this study was underpowered to reject the null hypothesis for specific complications, potentially leading to a type II error. As expected, baseline characteristics between groups were markedly different, which could have resulted in a selection bias. To minimize this potential bias, univariate and multivariate regression analyses were performed to assess confounding factors among baseline characteristics and found no difference in outcomes. The asymmetrical distribution of cases between the two institutions (58% of primary and 89% of revisional cases were performed in one site) could have led to a selection bias as well. Another limitation is the relatively low percentage of patients with follow-up greater than 12 months (approximately 50% in each group). Low postoperative follow-up percentages have been previously reported in the bariatric population [38], and this study is unfortunately no exception. Since the primary outcome of this study was postoperative complications and given a follow-up of at least 3 months for all patients, the authors believe that only late postoperative complications such as marginal ulcer or internal hernia could have potentially been missed for some patients. This possibility should not significantly change the main conclusions of this article. Ideally, a randomized clinical study should be performed to assess a potential advantage of robotically assisted versus laparoscopic surgery in patients undergoing revisional RYGB. The feasibility of such a study is however very limited due to the difficulty of developing equivalent expertise in robotically assisted and laparoscopic revisional bariatric surgery in a single center.

In conclusion, this study showed similar overall early and late complication rates between primary and revisional RAL RYGB, with higher rates of gastrojejunal strictures and readmissions for oral intolerance in the revisional group. Despite the study being underpowered to detect a statistically significant difference between groups in specific complications such as gastrointestinal leak rates, these results suggest that the higher morbidity reported in the literature in patients undergoing laparoscopic revisional RYGB can potentially be decreased, if not brought down, to the low morbidity seen in primary procedures, when using the robotic approach. Further prospective studies are needed to confirm these results.
